# Supplementary material for: Effect of Surfactants on the Binding Properties of a Molecularly Imprinted Polymer
Source: Polymers (Basel). 2022 Nov 30;14(23):5210. doi: 10.3390/polym14235210 (PMC9741244; doi:10.3390/polym14235210)
Supplement: Supplementary file 1 [file polymers-14-05210-s001.zip › polymers-2036133-supplementary.pdf]

Supplementary materials

Table S1: Tween 20, binding affinity values (K_{eq}) ± 1 standard error (in 10^3 mol l^{-1}) measured for 2,4,5-T on MIP & NIP and for 2,4-D on MIP

**2,4,5-T (on MIP)**	**Molar fraction of MeCN in water**				
	**0.00**	**0.24**	**0.42**	**0.66**	**0.81**
**no Tween 20**	80 ± 9	61 ± 5	78 ± 5	189 ± 3	830 ± 8
**Tween 20 0.81 mM**	77 ± 1	47 ± 3	69 ± 1	184 ± 1	749 ± 2
**Tween 20 4.10 mM**	61 ± 1	44 ± 3	57 ± 1	170 ± 1	709 ± 3
**Tween 20 8.10 mM**	56 ± 1	41 ± 1	46 ± 3	159 ± 1	649 ± 4
**Tween 20 20.40 mM**	53 ± 3	36 ± 2	43 ± 3	152 ± 1	622 ± 4

**2,4-D (on MIP)**	**Molar fraction of MeCN in water**				
	**0.00**	**0.24**	**0.42**	**0.66**	**0.81**
**no Tween 20**	56 ± 3	38 ± 2	47 ± 3	97 ± 2	431 ± 25
**Tween 20 0.81 mM**	52 ± 1	30 ± 1	42 ± 1	94 ± 1	364 ± 3
**Tween 20 4.10 mM**	47 ± 1	28 ± 1	39 ± 1	90 ± 1	358 ± 1
**Tween 20 8.10 mM**	42 ± 1	25 ± 2	32 ± 1	84 ± 1	351 ± 1
**Tween 20 20.40 mM**	37 ± 1	23 ± 1	28 ± 1	79 ± 1	324 ± 2

**2,4,5-T (on NIP)**	**Molar fraction of MeCN in water**				
	**0.00**	**0.24**	**0.42**	**0.66**	**0.81**
**no Tween 20**	39 ± 7	14 ± 3	25 ± 12	101 ± 8	398 ± 7
**Tween 20 0.81 mM**	21 ± 1	10 ± 1	20 ± 1	70 ± 1	264 ± 1
**Tween 20 4.10 mM**	15 ± 1	8 ± 3	17 ± 2	68 ± 4	254 ± 6
**Tween 20 8.10 mM**	15 ± 1	8 ± 2	15 ± 1	66 ± 1	255 ± 4
**Tween 20 20.40 mM**	15 ± 1	7 ± 1	13 ± 1	63 ± 1	250 ± 6

Table S2: SDS, binding affinity values (K_{eq}) ± 1 standard error (in 10^3 mol l^{-1}) measured for 2,4,5-T on MIP & NIP and for 2,4-D on MIP

**2,4,5-T (on MIP)**	**Molar fraction of MeCN in water**				
	**0.00**	**0.24**	**0.42**	**0.66**	**0.81**
**no SDS**	80 ± 9	61 ± 5	78 ± 5	189 ± 3	830 ± 8
**SDS 1.73 mM**	30 ± 1	6 ± 1	9 ± 1	31 ± 1	128 ± 2
**SDS 3.46 mM**	21 ± 5	7 ± 1	17 ± 1	27 ± 1	105 ± 2
**SDS 5.19 mM**	13 ± 2	5 ± 1	10 ± 1	24 ± 1	90 ± 1
**SDS 6.92 mM**	9 ± 1	4 ± 1	8 ± 4	20 ± 1	75 ± 2

**2,4-D (on MIP)**	**Molar fraction of MeCN in water**				
	**0.00**	**0.24**	**0.42**	**0.66**	**0.81**
**no SDS**	56 ± 3	38 ± 2	47 ± 3	97 ± 2	431 ± 25
**SDS 1.73 mM**	16 ± 1	7 ± 1	14 ± 1	19 ± 1	70 ± 2
**SDS 3.46 mM**	13 ± 1	5 ± 1	9 ± 1	14 ± 1	57 ± 1
**SDS 5.19 mM**	8 ± 1	4 ± 2	6 ± 1	12 ± 1	56 ± 1
**SDS 6.92 mM**	7 ± 1	3 ± 1	4 ± 1	10 ± 1	50 ± 1

**2,4,5-T (on NIP)**	**Molar fraction of MeCN in water**				
	**0.00**	**0.24**	**0.42**	**0.66**	**0.81**
**no SDS**	39 ± 7	14 ± 3	25 ± 12	101 ± 8	398 ± 7
**SDS 1.73 mM**	3 ± 1	2 ± 1	7 ± 1	10 ± 1	59 ± 1
**SDS 3.46 mM**	2 ± 1	1 ± 1	3 ± 1	8 ± 1	50 ± 6
**SDS 5.19 mM**	1 ± 1	1 ± 1	2 ± 1	7 ± 1	52 ± 1
**SDS 6.92 mM**	2 ± 1	1 ± 1	2 ± 1	5 ± 1	52 ± 2

Table S3: CTAB, binding affinity values (K_{eq}) ± 1 standard error (in 10^3 mol l^{-1}) measured for 2,4,5-T on MIP & NIP and for 2,4-D on MIP

**2,4,5-T (on MIP)**	**Molar fraction of MeCN in water**				
	**0.00**	**0.24**	**0.42**	**0.66**	**0.81**
**no CTAB**	80 ± 9	61 ± 5	78 ± 5	189 ± 3	830 ± 8
**CTAB 1.37 mM**	21 ± 1	9 ± 1	17 ± 1	20 ± 3	299 ± 2
**CTAB 2.74 mM**	22 ± 4	9 ± 1	12 ± 1	38 ± 2	187 ± 5
**CTAB 4.12 mM**	19 ± 1	6 ± 1	9 ± 2	34 ± 2	182 ± 12
**CTAB 5.90 mM**	16 ± 1	6 ± 1	8 ± 1	26 ± 1	153 ± 3

**2,4-D (on MIP)**	**Molar fraction of MeCN in water**				
	**0.00**	**0.24**	**0.42**	**0.66**	**0.81**
**no CTAB**	56 ± 3	38 ± 2	47 ± 3	97 ± 2	431 ± 25
**CTAB 1.37 mM**	16 ± 2	11 ± 1	14 ± 3	29 ± 1	249 ± 107
**CTAB 2.74 mM**	16 ± 1	6 ± 1	9 ± 1	30 ± 1	165 ± 7
**CTAB 4.12 mM**	15 ± 1	4 ± 1	6 ± 1	27 ± 3	15 ± 7
**CTAB 5.90 mM**	14 ± 3	4 ± 1	7 ± 1	19 ± 1	136 ± 7

**2,4,5-T (on NIP)**	**Molar fraction of MeCN in water**				
	**0.00**	**0.24**	**0.42**	**0.66**	**0.81**
**no CTAB**	39 ± 7	14 ± 3	25 ± 12	101 ± 8	398 ± 7
**CTAB 1.37 mM**	5 ± 2	4 ± 3	5 ± 2	22 ± 2	102 ± 6
**CTAB 2.74 mM**	4 ± 1	2 ± 1	3 ± 1	17 ± 2	88 ± 1
**CTAB 4.12 mM**	4 ± 1	1 ± 1	2 ± 2	13 ± 1	81 ± 20
**CTAB 5.90 mM**	3 ± 1	1 ± 1	1 ± 1	14 ± 2	76 ± 1
